# For the Information of Dentists

**Published:** 1879-04

**Authors:** 


### FOR THE INFORMATION OF DENTISTS.

Baltimore, March 8, 1879.

In the U. S. Circuit Court to-day in Baltimore, his Honor, Judge Bond, decided that it was not necessary for him to hear the evidence in the cases before him of the Goodyear Dental Vulcanite Co., against Dentists, inasmuch as the validity of the Cummings patent had been affirmed in this judicial district by his Honor, Judge Giles.

He therefore granted the decree against the defendants on the bill, answer and replication,—without the reading of the evidence or of either complainants or defendants.

